# Pyrophosphate-Mediated Iron Acquisition from Transferrin in *Neisseria meningitidis* Does Not Require TonB Activity

**DOI:** 10.1371/journal.pone.0107612

**Published:** 2014-10-07

**Authors:** Francis Biville, Christophe Brézillon, Dario Giorgini, Muhamed-Kheir Taha

**Affiliations:** Unité des Infections Bactériennes invasives, Département Infection et Epidémiologie, Institut Pasteur, Paris, France; Centre National de la Recherche Scientifique, Aix-Marseille Université, France

## Abstract

The ability to acquire iron from various sources has been demonstrated to be a major determinant in the pathogenesis of *Neisseria meningitidis*. Outside the cells, iron is bound to transferrin in serum, or to lactoferrin in mucosal secretions. Meningococci can extract iron from iron-loaded human transferrin by the TbpA/TbpB outer membrane complex. Moreover, *N. meningitidis* expresses the LbpA/LbpB outer membrane complex, which can extract iron from iron-loaded human lactoferrin. Iron transport through the outer membrane requires energy provided by the ExbB-ExbD-TonB complex. After transportation through the outer membrane, iron is bound by periplasmic protein FbpA and is addressed to the FbpBC inner membrane transporter. Iron-complexing compounds like citrate and pyrophosphate have been shown to support meningococcal growth *ex vivo*. The use of iron pyrophosphate as an iron source by *N. meningitidis* was previously described, but has not been investigated. Pyrophosphate was shown to participate in iron transfer from transferrin to ferritin. In this report, we investigated the use of ferric pyrophosphate as an iron source by *N. meningitidis* both *ex vivo* and in a mouse model. We showed that pyrophosphate was able to sustain *N. meningitidis* growth when desferal was used as an iron chelator. Addition of a pyrophosphate analogue to bacterial suspension at millimolar concentrations supported *N. meningitidis* survival in the mouse model. Finally, we show that pyrophosphate enabled TonB-independent *ex vivo* use of iron-loaded human or bovine transferrin as an iron source by *N. meningitidis.* Our data suggest that, in addition to acquiring iron through sophisticated systems, *N. meningitidis* is able to use simple strategies to acquire iron from a wide range of sources so as to sustain bacterial survival.

## Introduction

*Neisseria meningitidis* (*Nm*) is found exclusively in humans, and although it is frequently present in the nasopharynx of asymptomatic carriers, it may be the causative agent of life-threatening invasive infections such as septicemia and meningitis [1]. Ability to acquire iron from various sources has been demonstrated to be a major determinant in the pathogenesis of *Nm* [2]. In mammals, iron sequestration is the main form of nutritional immunity [3], [4]. Obtaining iron required for bacterial growth is a challenge, since 99.9% of total body iron is sequestered inside the cells [5]. Outside the cells, iron is bound to transferrin in the serum or to lactoferrin in mucosal secretions [2]. Another iron source in mammals is heme, mainly contained in hemoproteins like hemoglobin. When freed after erythrocyte lysis, most hemoglobin is bound by haptoglobin. Hemoglobin degradation allows the release of heme that is sequestered by hemopexin to prevent its toxicity [5]. Bacterial acquisition of iron in mammals requires the activity of transport systems allowing uptake of iron and/or heme bound to proteins. In *Nm*, the HmbR [6] and HpuAB outer membrane transport systems [7] allow the bacteria to use heme-loaded proteins as a heme source. HmbR and HpuAB systems differ according to their substrate specificity. HmbR can obtain heme from hemoglobin with better efficiency for human hemoglobin [6]. In contrast, HpuAB not does not exhibit specificity toward the human forms of its two substrates, characterized as hemoglobin and haptoglobin-hemoglobin complexes [8]. *Nm* strains express HmbR, HpuAB or both systems [9]. Most invasive strains express HmbR alone or both heme uptake systems, as reported in isolates of the hyperinvasive genotype ST-11 [9]. Strains expressing only the HpuAB heme transport system were mostly described as carriage strains [9]. The periplasmic heme binding protein and the inner membrane heme transporter are not yet identified. Inside the cytoplasm, heme is degraded by HemO, a bacterial heme oxygenase, thus allowing the release of iron [10].

The main source of iron in blood is iron-loaded transferrin. Iron is extracted from iron-loaded human transferrin by the TbpA/TbpB outer membrane complex [11]. Also, *Nm* expresses the LbpA/LbpB outer membrane complex, which can extract iron from iron-loaded human lactoferrin [12]. After transportation through the outer membrane, iron is bound by the periplasmic protein FbpA and directed to the FbpBC inner membrane transporter [13]. Most of heme and iron outer membrane transport systems require energy provided by the ExbB-ExbD-TonB system [14]. TonB independent iron transport processes were also reported. [15], [16]. Alongside the two systems allowing the obtaining of iron contained in human protein, *Neisseriae* genomes encode systems enabling uptake of free iron. The transport of iron-loaded xenosiderophores has been investigated in *Neisseria gonorrhoeae* [17]. Iron-loaded xenosiderophores are transported by the TonB-dependent outer membrane transporter FetA [18], sent by FbpA to the inner membrane FbpBC transporter and degraded inside the cytoplasm to allow iron release [15]. TonB-independent transport of xenosiderophores through the outer membrane has been described in *N. gonorrhoeae,* but the mechanism remains hypothetical [17]. In contrast, the role of the FbpABC inner membrane ABC transporter in TonB-independent use of enterobactin, salmochelin and other xenosiderophores has been clearly demonstrated [15]. The absence of siderophore biosynthesis was reported for *Nm* [19]. Only the use of a ferrated form of three dihydroxamate siderophores (schizokinen, arthrobactin, aerobactin) can stimulate growth of *Nm* [20]. Recently, the binding of Ferric enterobactin by the factor H binding protein was described [21].

Iron-complexing compounds like citrate and pyrophosphate have been shown to support *Nm* growth *ex vivo* [19]. The use of iron pyrophosphate as an iron source by *N. meningitis* was described, but not investigated. Pyrophosphate-dependent use of iron was investigated in *Escherichia coli* [22], [23]. In that bacterium, pyrophosphate facilitates the enterobactin-dependent iron uptake process [24]. In the absence of enterobactin, pyrophosphate acts as an iron chelator and strongly inhibits *E. coli* growth [25]. Also, pyrophosphate was shown to participate in iron transfer from transferrin to ferritin [26]. This report aimed to investigate the mechanism that allows use of ferric pyrophosphate as an iron source and its impact on meningococcal virulence.

## Materials and Methods

### Ethics statement

This study was carried out in strict accordance with the European Union Directive 2010/63/EU (and its revision 86/609/EEC) on the protection of animals used for scientific purposes. Our laboratory has the administrative authorization for animal experimentation (Permit Number 75–1554) and the protocol was approved by the Institut Pasteur Review Board that is part of in the Regional Committee of Ethics of Animal Experiments of the Paris region (CETEA 2013-0190).

### Bacterial strains and plasmids

Bacterial strains and plasmids used in this study are listed in Table 1.

**Table 1:** Strains and plasmids used in this study.

*Escherichia coli strain*	Genotype	Origin
Xl1 blue	F*^−^ supE44 hdsR17 recA1 endA1 gyrA46 thi relA1 lac^−^ F’ proAB^−^ lacI^q^ lacZΔM15 Tn10* (Tet^R^)	Laboratory collection
***Nm*** ** strains**	**Genotype**	**Origin**
2C4.3	wild-type strain	[59]
2C4.3*::lux*	2C4.3*::lux*, Km^R^	This work
2C4.3 *ΔtonB::Ery*	2C4.3 *ΔtonB::Ery*, Ery^R^	This work
2C4.3 *ΔfbpABCD::Ery*	2C4.3 *ΔfbpABCD::Ery*,Ery^R^	This work
2C4.3 *ΔporA::Ery*	2C4.3 *ΔporA::Ery*, Ery^R^	This work
2C4.3 *ΔporB::Ery*	2C4.3 *ΔporB::Ery,* Ery^R^	This work
2C4.3*::lux ΔtonB::Ery*	2C4.3*::lux ΔtonB::Ery*, Km^R^, Ery^R^	This work
**Plasmids**	**Antibiotic resistance**	**Origin**
pXen-13	Amp^R^	Xenogen
pTE-KM	Amp^R^, Km^R^	[29]
pDG33	Amp^R^	This work
pDG34	Amp^R^, Km^R^	This work

### Media and growth conditions

Human (Sigma; ref: T4132) and bovine (Sigma; ref: T1283) transferrin were prepared in water at 0.25 mM final concentration, filter-sterilized and stored at −20°C. Bovine hemoglobin (Sigma; ref: H2500) was dissolved in 100 mM NaCl, filter-sterilized and stored at −20°C. The hemoglobin concentration was calculated on the basis of the heme monomer. Tetrasodium pyrophosphate (Sigma; ref: P8010) was prepared at a 200 mM final concentration in water, buffered at pH 7 with HCl, filter-sterilized and stored at room temperature. Imdidodiphosphate (Sigma; ref: 10631) and methylenediphosphonic acid (Sigma; ref: M9508) were prepared according to the same protocol and stored at −20°C. Iron pyrophosphate (Sigma; ref: P6526) was prepared in water at a 10 mM final concentration, filter-sterilized and stored at room temperature. Desferal (Sigma; ref: D9533) was prepared in water at 15 mM, filter-sterilized and stored at −20°C. All solutions were filter-sterilized using 0.20 µm Millipore filters. *Nm* strains were grown on GCB agar plates supplemented with Kellogg supplement solution [27]. To create iron depletion, supplement S2 was substituted for desferal (30 µM final concentration). When required, kanamycin (Kan) and erythromycin (Ery) were added at 50 µg/ml, and 2 µg/ml respectively. *Nm* strains were grown at 37°C under a 5% CO_2_ atmosphere. *E*. *coli* strains were grown on LB medium [28] at 37°C. Solid media agar contained 1.5% agar.

### Use of iron source assays

To evaluate the effect of mutation of the *Nm* capacity to use various iron sources, strains were first isolated on GCB plates supplemented with S1 and S2 complements and grown for 18 h at 37°C in the presence of 5% CO_2_. Bacteria were isolated on the test plates and incubated for 18 h at 37°C in the presence of 5% CO_2_. Iron-depleted GCB plates (see above) were supplemented with the tested iron sources.

### Invasion assays in mice

*Nm* tested strains were grown on GCB plates for 18 h at 37°C under a 5% CO_2_ atmosphere. Bacteria collected from one plate were suspended in physiological serum and the density of the cell suspension was adjusted to 2.5×10^6^ bacteria/ml. Four-hundred µl of the bacterial suspension were supplemented with 100-µl of the tested iron source, and the mixture was inoculated intraperitoneally into 7-week-old BalbC mice (Janvier). The number of viable bacteria before inoculation was then determined by plating serial dilutions on GCB plates. At t = 6 h, blood and intraperitoneal samples were collected, diluted in physiological serum and serial dilutions were plated on GCB plates supplemented with S1 and S2 and kanamycin (50 µg/ml). After 18 h incubation at 37°C under a 5% CO_2_ atmosphere, colonies were counted.

### Imaging of bioluminescence from animals

Mice were then anesthetized with a constant flow of 2.5% isoflurane mixed with oxygen, using an XGI-8 anesthesia induction chamber (Xenogen Corp.). The mice were maintained for at least 5 min. Bacterial infection images were acquired using an IVIS spectrum system (Xenogen Corp., Alameda, CA) according to instructions from the manufacturer. Analysis and acquisition were performed using Living Image 3.1 software (Xenogen Corp.). Images were acquired using a 1 min integration time with a binning of 16. All other parameters were held constant. Quantifying was performed using the photons per second emitted by each mouse.

### Genetic techniques

*Nm* was transformed using linear 3-partner PCR fragments obtained as described below. *Nm* strains were grown on GCB plates for 18 h at 37°C under a 5% CO_2_ atmosphere. Bacteria collected from one plate were suspended in GCB medium completed with S1 and S2 supplements and MgCl2 at a 5 mM final concentration (GCBMg medium). Bacterial density was adjusted at OD_600_∶1. Three hundred microliters of the bacterial suspension were placed inside a well of a 24-well multiwell plate (Falcon), supplemented with a PCR fragment (100 to 500 ng) and incubated for 30 min at 37°C under a 5% CO_2_ atmosphere. The mixture was supplemented with 700 µl of GCBMg medium and incubated for 5 h at 37°C under a 5% CO_2_ atmosphere. One-hundred and 500 µl samples of the mixture were plated on GCB complete medium supplemented with selective antibiotic and incubated for 18 h at 37°C under a 5% CO_2_ atmosphere. Six clones were isolated on selective medium, screened using PCR and positive clones were stored at −80°C in complete GCB medium supplemented with glycerol (20% final concentration). For *fbpABC* mutants, GCB supplemented with S1 Kellogg supplement solution [27], bovine hemoglobin (10^−6^ M) and erythromycin was used as selective medium.

### DNA manipulations

DNA fragments were amplified from chromosomal *Nm* strain 2C4.3 in a Hybaid PCR thermocycler using Phusion DNA polymerase (Finnzymes). Restriction, modification and ligation were carried out according to the manufacturer’s recommendations. Purification of DNA fragments from the PCR reaction, the restriction reaction and agarose gels was performed using the Macherey-Nagel NucleoSpin Extract II kit.

### Construction of the 2C4.3*::lux* strain

Plasmid pXen-13 (Xenogen Corp., Alameda, CA) containing the *Photorhabdus luminescens luxCDABE* operon was modified by insertion of an *Nm-*specific promoter sequence. To express the *luxCDABE* operon under the PproB meningococcal promoter *Nm*, a 600 bp promoter sequence of the *porB* gene (PporB) from strain 2C4.3 was amplified using primers PorB3 and PorB4 (Table 2) and cloned into a *Bam*HI site upstream of the *luxCDABE* operon after Kleenow filling. The generated plasmid was named pDG33. The fragment encompassing the *luxCDABE* cassette and the *porB* promoter was extracted by digesting pDG33 with KpnI and SacI restriction enzymes and inserted into the *Bam*HI site of plasmid pTE-KM [29], upstream from the kanamycin resistance cassette *aph3’*. In the resulting vector, named pDG34, the PporB-*luxCDABE*-*aph3’* was flanked by the meningococcal *pilE* gene and, 120 bp downstream, by the *pilE* gene to facilitate chromosomal integration upon transformation.

**Table 2:** Primers used in this study.

Name	Sequence 5′-3′
Eram 1	**GCAAACTTAAGAGTGTGTTGA**
Eram 3	**AAGCTTGCCGTCTGAATGGGACCTCTTTAGCTTCTTGG**
PorB3	**GGTGCTGAAGCACCAAGTGA**
PorB4	**GGCAATCAGGGATTTTTTCA**
TonBAmtAmt	**ACAGAATCGCATTGATCAGAATACCG**
TonBAmtAvlEry	**TCAACACACTCTTAAGTTTGCTAAAATTCGTTCTTTATCCATAATTC**
TonBAvlAmtEry	**CCAAGAAGCTAAAGAGGTCCCATTCAGACGGCAAGCTTAGTCCCCGTCAAGTTTGAATTGAATTAG**
TonBAvlAvl	**TCAGCTATCCTTTTGATTAAGCAGGC**
FbpABCAmtAmt	**GACGCATTGAGAAAAGAAATCCGCCACCTCCAAT**
FbpABCAmtAvlEry	**TCAACACACTCTTAAGTTTGCTCAGGGCTGCGGCAAGCAGTGCGTATCGGATAG**
FbpABCAvlAmtEry	**CCAAGAAGCTAAAGAGGTCCCATTCAGACGGCAAGCTTACGGTCCCGCCCTGTTCTTCCCCGGAAATACC**
FbpABCAvlAvl	**ATGGCGGCGGCTTGGACGGTGCGGGGTTCTG**
PorAAmtAmt	**TGTAGATGCCCGACGGTCTTTATAGCGG**
PorAAmtAvlEry	**TCAACACACTCTTAAGTTTGCTGTAAATTTGATAAAAACCTAAAAACATCG**
PorAAvlAmtEry	**CCAAGAAGCTAAAGAGGTCCCATTCAGACGGCAAGCTTGAAGCGGATAGCTTTGTTTTTGACGGCTCG**
PorAAvlAvl	**TATATTCCGCCATCTCTAAGATTTACAGCG**
PorBAmtAmt	**ATCGGTTCCGTACTATTTGTACTGTCTGCG**
PorBAmtAvlEry	**TCAACACACTCTTAAGTTTGCTCGTAGTTAAGAAATTTAAGCAGACCTAAC**
PorBAvlAmtEry	**CCAAGAAGCTAAAGAGGTCCCATTCAGACGGCAAGCTTTCTGAAAAGATTGGTATCAACAAAAAGCCTG**
PorBAvlAvl	**ACAGATAGTAGGGAACCGATTCACTTGGTG**

### Construction of knockout mutants in *Nm*

Non-polar mutations that delete entire genes were created by allelic exchange with the non-polar Ery gene cassette. For knockout genes in *Nm*, the methods already described require the use of *E. coli* to clone, in plasmids, *Nm* DNA fragments containing a gene of interest, disrupted by insertion of a cartridge expressing antibiotic resistance [30]. These methods require cloning steps and are subordinated to the stability of the recombinant plasmids and their absence of toxicity when introduced into *E. coli*. To avoid the use of cloning steps, we directly introduced into *Nm* the disrupted genes contained in the DNA fragment obtained using a two-step PCR procedure. The two-step PCR procedure was used to produce a PCR product in which the Ery gene cassette is flanked by arms of about 500 to 1,000 bp, corresponding to sequences upstream from the start codon and downstream from the stop codon of the gene of interest. The erythromycin cartridge was amplified from plasmid pMGC20 [31] using Eram1 and Eram3 as primers (Table 2). The primers used for *tonB* were TonBAmtAmt and TonBAmtAvlEry for the upstream region and TonBAvlAmtEry and TonBAvlAvl for the downstream region (Table 2). For *porA*, the primers used were PorAAmtAmt and PorAAmtAvlEry for the upstream region and PorAAvlAmtEry and PorAAvlAvl for the downstream region (Table 2). For *porB*, the primers used were PorBAmtAmt and PorBAmtAvlEry for the upstream region and PorBAvlAmtEry and PorBAvlAvl for the downstream region (Table 2). To delete *fbpABC*, the primers used were FbpABCAmtAmt and FbpABCAmtAvlEry for the upstream region and FbpABCAvlAmtEry and FbpABCAvlAvl for the downstream region (Table 2). For each gene of interest, the sequence of the 5′ end of the reverse primer used to amplify the upstream region was anti-parallel to the 5′end of Eram1 primer and the sequence of the 5′ end of the forward primer used to amplify the downstream region was anti-parallel to the 5′ Eram3 primer. For each gene of interest, a 1 µl sample of upstream and downstream regions was mixed with 1 µl of the erythromycin cartridge and the mixture was amplified using primers TonBAmtAmt and TonBAvlAvl for *tonB*, PorAAmtAmt and PorAAvlAvl for *porA*, PorBAmtAmt and PorBAvlAvl for *porB* and FbpABCAmtAmt and FbpABCAmtAvl for *fbpABC*. Various amounts of the three partners PCR fragments were introduced into *Nm* using the transformation method described above. Correct localization of the chromosomal insertion was checked by PCR amplification using cat primers Eram1 and Eram3, in combination with primers TonBAvlAvl and TonBAmtAmt for *tonB* disruption, PorAAvlAvl and PorAAmtAmt for *porA* deletion, PorBAvlAvl and PorBAmtAmt for *porB* deletion or FbpABCAvltAvl and FbpABCAmtAmt for *fbpABC* deletion.

### Iron binding assay

The ability of desferal, pyrophosphate and its structural analogues to bind iron Fe^3+^ was visualized with a classical assay used to quantify siderophores in solution [32].

### Statistical analysis

Data are expressed as the mean ± SD of 5 samples, and the reproducibility was confirmed at least in three separate experiments. Statistical analysis were performed using two-way unpaired Student’s *t*-test and considered significant if *P*<0.05.

## Results

### *Ex vivo* use of ferric pyrophosphate as an iron source

**Figure 1:**
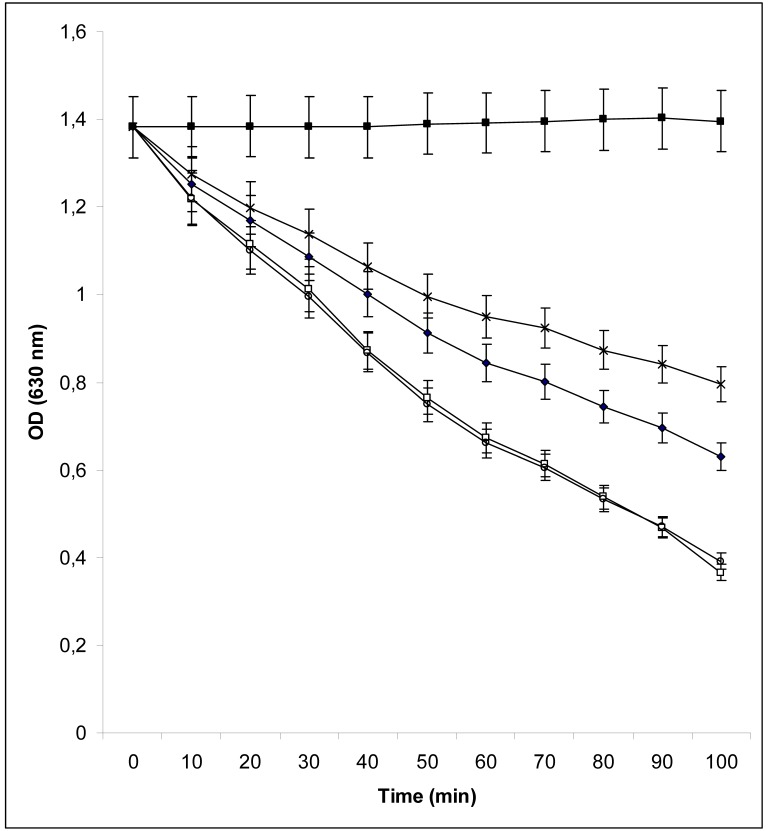
Assay of the ability of desferal, pyrophosphate (PPi), methylenediphosphonic acid (PcP) and imidodiphosphate (PnP) to bind iron. 150 nanomoles of desferal (♦), PPi (○), PcP (□), or PnP (X) were added to a 1 ml mix (1/4 V/3/4 V) of distilled water and CAS assay solution [32] at room temperature. Every 10 min for 60 min absorbance was measured at 630 nm. (▪): No agent added. The experiment was repeated three times. A representative result is presented.

In a first set of experiments, we investigated the *ex vivo* use of ferric pyrophosphate as an iron source by *Nm* strain 2C4.3. The tested strain was cultured on GCB medium supplemented with S1 complement and desferal 15 µM or 30 µM to create iron depletion. On this medium, no growth of the *Nm* 2C4.3 strain was observed. The addition of iron pyrophosphate led to growth restoration (Table 3). The minimal concentration of iron pyrophosphate required for growth on GCB iron-depleted medium was 15 µM (Table 3). In iron pyrophosphate, the iron content was about one-tenth of the iron pyrophosphate compound in weight. In spite of the presence of desferal used as a chelator, *Nm* was able to use iron pyrophosphate as an iron source. This suggested that the affinity of pyrophosphate for iron was higher than that of desferal. This hypothesis was strengthened by comparing the ability of pyrophosphate and desferal to induce a color change in an iron dye complex used to detect and quantify siderophores [32]. This ability was related to the capacity to bind iron and release free dye [32]. As seen in Figure 1, pyrophosphate induced a strong color change at 630 nm, reflecting its ability to bind iron [24]. In contrast, with desferal, the free dye release occurred much more slowly (Figure 1).

**Table 3:** Use of iron pyrophosphate (FePPi), FeNo3 and FeCl3 as iron sources by *Nm.*

Desferal 15 µM					Desferal 30 µM			
µM	20	15	10	5	20	15	10	5
**FePPi**	**+++**	**+++**	−	−	**+++**	**+++**	−	−
**FeNo3**	**+++**	−	−	−	**+**	−	−	−
**FeCl3**	**+++**	−	−	−	−	−	−	−

Experiments were repeated three times. Representative results are presented. **+++**: large colonies (1 to 1.5 mm diameter); **+**: small colonies (<0.5 mm diameter); −: no growth.

### The iron pyrophosphate transport pathway in *Nm*

**Figure 2:**
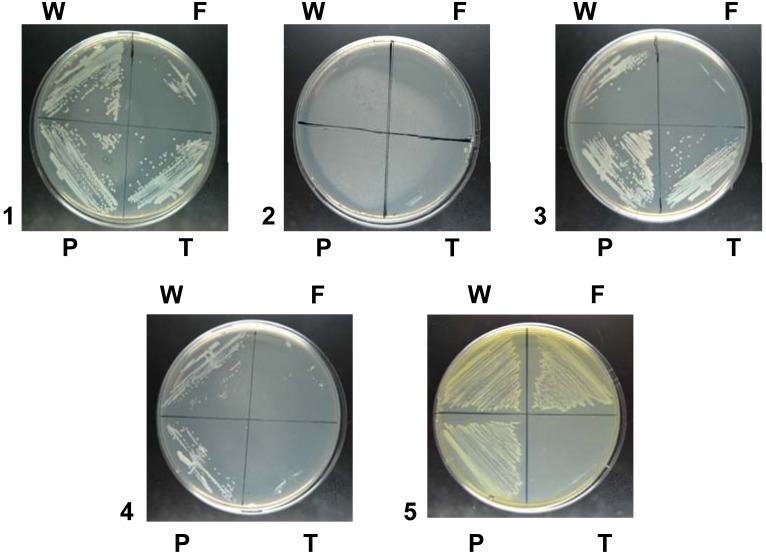
Mutations impairing iron pyrophosphate (FePPi) uptake in *Nm*. Strains 2C4.3 (W), 2C4.3 *ΔporA* or 2C4.3 *ΔporB* (P) and 2C4.3 *ΔtonB* (T) were isolated on a GCB plate supplemented with S1 and S2 complements and grown for 18 h at 37°C in the presence of 5% CO_2_. For strain 2C4.3*ΔfbpABC* (F), GCB medium was supplemented with Kellogg supplement 1 solution [27] and human hemoglobin was added at a 5×10^−6^ M final concentration. All tested strains were then isolated on GCB medium (1), GCB medium with desferal (2), GCB medium with desferal and FePPi 15 µM (3), GCB medium with desferal and human transferrin 5×10^−6^ M (4), GCB medium with desferal and hemoglobin 5×10^−6^ M (5). Bacteria were isolated on the test plates and incubated for 18 h at 37°C in the presence of 5% CO_2_. The experiment was repeated three times. Representative results are presented.

In order to be used by the bacteria, the iron source must be transported through the outer membrane, the periplasm and the inner membrane. Outer membrane transport of iron and heme primarily involves transporters requiring the presence of the ExbB-ExbD-TonB complex as an energy provider [33]. We first checked for the effect of *tonB* disruption upon the ability of *Nm* to use iron pyrophosphate as an iron source. As shown in Figure 2, *tonB* disruption did not impair the use of iron pyrophosphate as an iron source. In contrast, the use of iron-loaded human transferrin and hemoglobin as an iron source was abolished in the *Nm tonB* mutant. Similarly, disruption of *porA* or *porB* structural genes encoding for the *Nm* major porins [34] had no effect on the use of iron pyrophosphate as an iron source (Figure 2). The inner membrane FbpABC transporter was shown to be required for the use of transferrin and xenosiderophores as iron sources [35], [15]. We thus tested the effect of *fbpABC* disruption of the capacity of *Nm* to use iron pyrophosphate as an iron source. As seen in Figure 2, *fpbABC* disruption abolished the use of iron pyrophosphate as an iron source.

### Exogenous pyrophosphate allows iron utilization in the presence of desferal

GCB medium can support *Nm* growth in the presence of supplement S1 and in the absence of supplement S2. Thus, iron traces present in the medium are sufficient for sustaining *Nm* growth. Addition of desferal at 15 µM or 30 µM abolished *Nm* growth on this medium. Addition of pyrophosphate, at 5 mM or higher, restored the growth of *Nm* on GCB S1 medium supplemented with desferal (Table 4). This result is in good agreement with the high affinity of pyrophosphate for iron [24]. The use of two structural analogues of pyrophosphate strengthened this conclusion. Imidodiphosphate and methylenediphosphonic acid were added to iron depleted GCB medium and growth of *Nm* was investigated. Our results demonstrated that methylenediphosphonic acid, similarly to pyrophosphate, allowed *Nm* growth on iron-depleted medium when added at a 5 mM final concentration (Table 4). In contrast, addition of imidodiphosphate did not support *Nm* growth on the same medium (Table 4). These results are in accordance with our results demonstrating that pyrophosphate and methylenediphosphonic acid, in contrast to imidodiphosphate, bind iron with higher affinity than desferal (Figure 1). Pyrophosphate and methylenediphosphonic-acid-dependent use of iron did not require TonB activity, but was abolished when *fbpABC* genes were disrupted (data not shown).

**Table 4:** Effect of pyrophosphate (PPi), methylenediphosphonic acid (PcP) and imidodiphosphate (PnP) on *Nm* growth on iron-depleted medium.

Desferal 15 µM					Desferal 30 µM			
mM	10	5	2	1	10	5	2	1
**PPi**	**+++**	**+++**	−	−	**+++**	**+++**	−	−
**PcP**	**+++**	**+++**	−	−	**+++**	**+++**	−	−
**PnP**	−	−	−	−	−	−	−	−

The experiment was repeated three times. Representative results are presented. **+++**: large colonies (1 to 1.5 mm diameter); −: no growth.

### Pyrophosphate enables TonB-independent use of transferrin as an iron source

**Figure 3:**
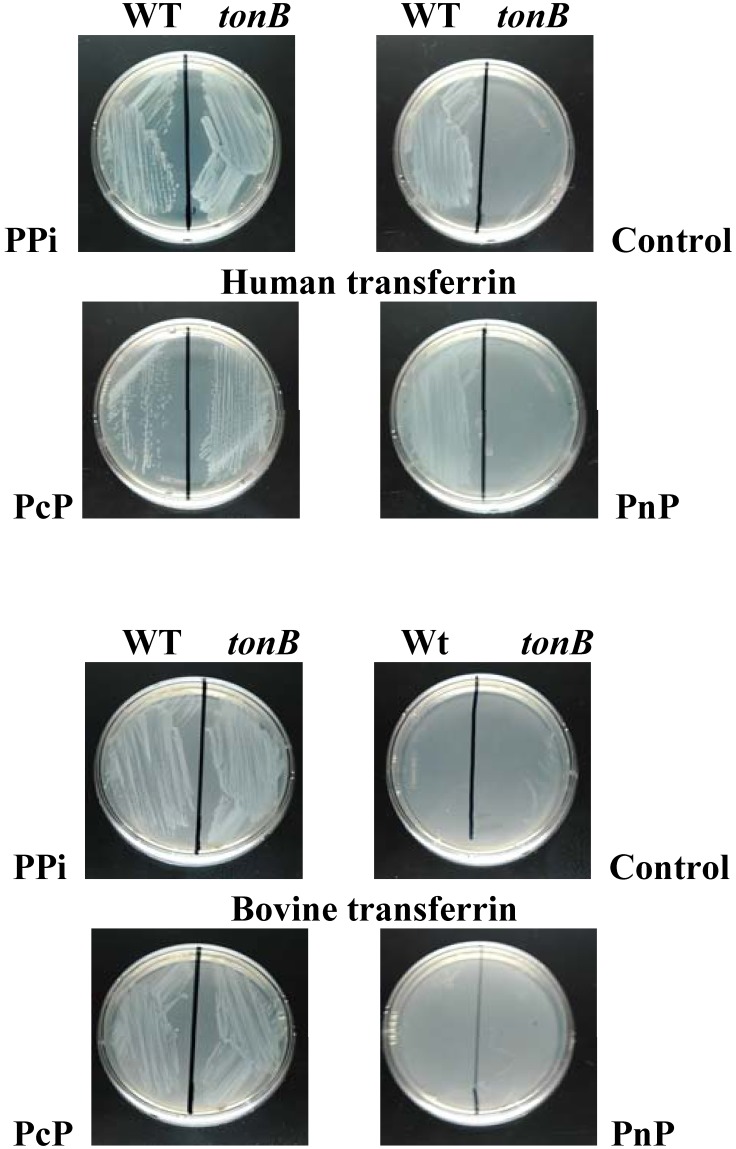
TonB-independent use of transferrin as an iron source. The tested strains were isolated on a GCB plate supplemented with S1 and S2 complements and grown for 18 h at 37°C in the presence of 5% CO_2_. GCB plates depleted for iron by addition of desferal were supplemented with human or bovine transferrin added at a 5 µM final concentration. When specified, PPi, PcP and PnP were added at a 1 mM final concentration. Bacteria were isolated on test plates and incubated for 18 h at 37°C in the presence of 5% CO_2_. The experiment was repeated three times. Representative results are presented.

In *Nm*, the use of transferrin as an iron source is restricted to human transferrin [2]. Other transferrins are not used by *Nm* as an iron source. Transportation of iron from human transferrin requires the activity of the TonB-dependent outer membrane transporter TbpAB [2]. *In vitro* experiments demonstrated the role of pyrophosphate as a mediator of iron transfer from transferrin to ferritin [36]. According to these results, pyrophosphate can bind iron loaded on transferrin and deliver it to ferritin [26]. Other authors demonstrated transfer from iron-loaded transferrin to pyrophosphate [37], [38]. The results described in these reports prompted us to check for the effect of pyrophosphate on the use of iron-loaded transferrin as an iron source. In a first set of experiments, we investigated the effect of pyrophosphate on the use of human transferrin and bovine transferrin as an iron source. As shown in Figure 3, in the absence of pyrophosphate, *Nm* used only human transferrin as an iron source. In contrast, in the presence of pyrophosphate, both bovine and human transferrins were iron sources for *Nm*. This cannot be explained by solubilization of contaminating iron, since the concentration of pyrophosphate used in this assay (1 mM) was not sufficient for supporting *Nm* growth in the presence of 30 µM desferal (Table 4). Since the TbpAB transport system exhibits absolute specificity for human transferrin, we hypothesized that the transport pathway used in the presence of pyrophosphate was independent of TbpAB activity. As a consequence, *tonB* disruption would not have an effect on the use of human or bovine transferrin in the presence of pyrophosphate. This was shown to be the case (Figure 3). Human and bovine transferrin as an iron source was also used in the presence of the two pyrophosphate analogues already tested in this report. As seen in Figure 3, imidodiphosphate addition did not alter the phenotype observed with wild type and *tonB* mutant strains. In contrast, similarly to pyrophosphate, methylenediphosphonic acid allowed TonB-independent use of human and bovine transferrin as iron sources (Figure 3).

### Effect of methylenediphosphonic addition upon *Nm* survival in the mouse model

**Figure 4:**
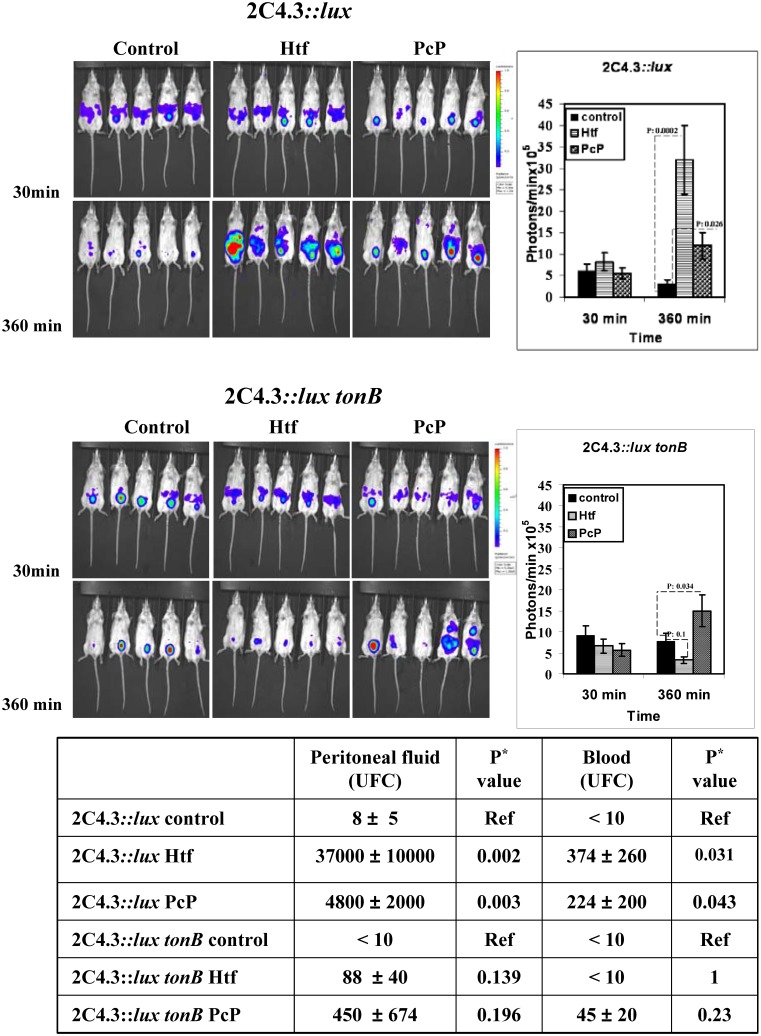
*Nm* growth in the mouse model in the presence of human transferrin (Htf) or methylenediphosphonic acid (PcP). The tested strains were isolated on a GCB plate supplemented with S1 and S2 complements and grown for 18 h at 37°C in the presence of 5% CO_2_. Bacteria were suspended in sterile physiological serum to obtain a cell density of 2.5×10^6^ bacteria/ml. When specified, 100 µl of the tested iron source were added to 400 µl of the bacterial suspension to obtain 0.05 mM for human transferrin and 5 mM for PcP. For the control experiment, 100 µl of physiological serum were added. For each experiment, the mixtures were injected intraperitoneally into five mice and bioluminescence was measured 30 min, and 360 min after injection, as described in Materials and methods. At t = 360 min, blood and peritoneal washes samples were taken, diluted in physiological serum and plated on GCB solid medium. After 18 h incubation at 37°C in the presence of 5% CO_2_, the colonies were counted. Data represent the means ± SD from 3 independent experiments of groups of five mice per time point in each experiment. Student’s *t*-test results were included in the figure and in the table. CFU: colony-forming unit.

In the absence of a usable iron source, *Nm* is cleared from mice very rapidly after intraperitoneal injection [39]. Addition of human transferrin to the bacterial suspension allows *Nm* to survive in mice [39]. Since the above results demonstrated that addition of pyrophosphate and methylenediphosphonic acid enabled the use of non-human transferrin as an iron source, we hypothesized that the addition of pyrophosphate and its structural analog, methylenediphosphonic acid, could promote the use of mouse transferrin as an iron source and enhance the survival of *Nm* in the mouse model. The addition of iron pyrophosphate (50 µM final concentration), pyrophosphate (5 mM final concentration) or imidodiphosphate (5 mM final concentration) had no effect on survival of *Nm* in mice (data not shown). In contrast, addition of 5 mM methylenediphosphonic acid, which is not degraded by inorganic pyrophosphatase [24], [40], increased significantly the ability of wild-type *Nm* to survive in the mice compared to control untreated mice (*p* = 0.026) (Figure 4). However, this effect of methylenediphosphonic acid on *Nm* growth was less prominent than that obtained by the addition of human transferrin (*p* = 0.0002) compared to control untreated mice (Figure 4). As evidenced in *ex vivo* assays, the effects of methylenediphosphonic acid were not abolished by *tonB* disruption. We therefore tested, in the mouse model (*in vivo*), the impact of *tonB* disruption on bacterial survival. As shown in figure 4, dynamic imaging result showed that *tonB Nm* mutant still showed significant better survival in mice treated with PcP compared to untreated control (*p* = 0.034). At the opposite, no more difference of survival *tonB Nm* mutant in human transferrin-treated mice compared to untreated control (*p* = 0.1) (Figure 4). We further study the survival of the wild type and the *tonB* mutant during the experimental infection in mice by bacterial counting from the peritoneal cavity and from the blood. The bacterial counts in blood and peritoneal cavity corroborated the results of dynamic imaging obtained with the wild type strain (Figure 4). For the *tonB* mutant, the results of the bacterial count in blood and peritoneal cavity are in a good accordance with the results of dynamic imaging when human transferrin was added (Figure 4). When methylenediphosphonic acid was added, a non significant trend for higher bacterial counts in the blood was observed (Figure 4). These data suggest that methylenediphosphonic acid enables a wide range of iron acquisition during experimental infection.

## Discussion

Iron acquisition by pathogenic *Neisseria* within the host is a major virulence trait. Bacteria employ specific receptors to obtain this transition metal from iron-containing proteins (transferrin, lactoferrin) in a TonB-dependent manner. However, tonB-independent pathways have been described. The mechanisms and significance of these pathways are not yet understood. We describe here a TonB independent iron transport process in *Nm*. This TonB-independent process allows *ex vivo* transportation of the iron pyrophosphate complex through the outer membrane. *In vitro*, pyrophosphate and methylenediphosphonic acid (a structural analogue of pyrophosphate) bind iron with higher affinity than desferal, and rescue *Nm* growth on plates in the presence of desferal in a TonB-independent manner. Iron-complexing compounds like citrate and pyrophosphate have been shown to support *Nm* growth *ex vivo* on culture plates [19], but their transport pathways have not been investigated. Using a rapid method, we built various mutants that enabled demonstrating the TonB-independent mechanism responsible for transport of iron pyrophosphate. *porA* or *porB* inactivation did not abolish the ability to use iron pyrophosphate as an iron source. Iron-loaded pyrophosphate could pass the outer membrane through both PorA and PorB porins. This hypothesis is in good agreement with identification of phosphate and ATP as PorB ligands [41]. Also, iron pyrophosphate can pass the outer membrane through a porin hypothesized to be responsible for the TonB independent use of xenosiderophores [17]. Thus, the transport pathway of iron pyrophosphate through the *Nm* outer membrane remains to be elucidated. FbpABC was shown to be responsible for the transport of iron pyrophosphate through the inner membrane. This complex was already shown to be required for iron transport through the inner membrane in *Nm* [35] and *N. gonorrhoeae* [13].

Pyrophosphate was shown to have a siderophore-like activity when ferritin was used as an iron source [19]. Moreover, pyrophosphate was shown to transfer iron from transferrin to ferritin [26]. Accordingly, our data obtained on plates demonstrate that pyrophosphate permits TonB-independent use of iron that is loaded from both human and bovine transferrin. In contrast, the acquisition of iron from transferrin through the TbpAB transporter is highly specific to human transferrin [11]. Indeed, it was previously shown that transgenic mice expressing human transferrin, or injection of iron-loaded human transferrin mice, leads to meningococcal growth in these animal models [39], [42]. We therefore explored, in the mouse model (*in vivo*), the significance of our finding concerning the role of iron pyrophosphate, pyrophosphate and its analogues on plates (*ex vivo*). Addition of pyrophosphate did not increase survival capacity in the mice in the absence of added human transferrin. Pyrophosphate degradation by inorganic pyrophosphatase [43]–[45] can explain this result. Addition of methylenediphosphonic acid increases survival of *Nm* in mice (Figure 4). This effect, also observed on a *tonB* mutant, suggests that TonB-independent transport of iron bound to methylenediphosphonic acid can support the growth of *Nm* in mice. Bacterial CFU counting revealed that *tonB* disruption decreased the ability of *N. meningitidis* to survive in the mice model in the presence of both PcP and human transferrin (Figure 4). The effect may be due to a decreased use of murine hemoglobin as an iron source in a TonB-dependent manner [6]. However, the decrease was prominent in the presence of human transferrin. Since iron-loaded transferrin is the main iron source in mice, we propose that methylenediphosphonic acid is able to obtain iron from mouse transferrin, as from bovine and human transferrin, and to form a ferric complex that can be transported through the outer membrane. According to the results obtained with dynamic imaging method, the use of mouse transferrin in the presence of methylenediphosphonic acid not requires the TonB activity. The effect of methylenediphosphonic acid addition on *Nm tonB* mutant survival inside the mice cannot be related to *tonB* reversion, since bacteria recovered from the intraperitoneal cavity and from blood were unable to use human transferrin and hemoglobin on plates. Taken together, the data in this report demonstrate *ex vivo* and *in vivo* pyrophosphate-mediated use of iron-loaded transferrin as iron sources. *Ex vivo* data clearly demonstrate that the pyrophosphate-mediated use of iron-loaded transferrin as an iron source not requires the TonB activity. Similarly to pyrophosphate-dependent iron uptake, other TonB-independent iron uptake processes have been described *ex vivo* in *Neisseria* [46], [15].

In *Escherichia coli*, pyrophosphate acts as an iron chelator in an *entF* strain that is unable to synthesize enterobactin, but is still able to produce dihydroxybenzoic acid [25]. This demonstrates that iron pyrophosphate cannot be used as an iron source in the absence of enterobactin in *E. coli*. In *Nm*, which was not demonstrated to produce siderophore, pyrophosphate addition counteracts the iron chelating of desferal, and iron pyrophosphate can be used as an iron source. The pore sizes of PorA (1.4 nm) [47] and PorB (1.6 nm) [48], [49] porins from *Nm* are close to those of OmpC (1,3 nm) and OmpF (1,4 nm) porins from *E. coli* [50], suggesting similar transport of iron pyrophosphate across the outer membrane. In *Neisseria*, the transport system responsible for transportation of iron pyrophosphate through the inner membrane was identified as FbpABC. This inner membrane transport system, was already demonstrated to be required for transport of iron from transferrin [35] and exogenous siderophores [15]. In FbpA, phosphate was identified as a synergistic anion allowing tight sequestration of iron [51]. Similarly to phosphate, pyrophosphate or methylenediphosphonic acid could play the role of a synergistic anion. This was suggested for pyrophosphate, and phosphatase activity was hypothesized for FbpA [51]. According to results obtained with methylenediphosphonic acid, this phosphatase activity is not required for the synergistic activity of pyrophosphate. In *E. coli*, that synthesizes siderophore, two periplasmic binding proteins and inner membrane transporters facilitate the transport of ferric-siderophore complexes. FhuD, the periplasmic protein responsible for directing ferric hydroxamate to the inner membrane FhuBC_2_ ABC transporter, also facilitates the transport of ferrichrome, coprogen, ferrioxamine B and aerobactin [52]. FepB binds ferric enterobactin and enterobactin in the periplasm and directs it to the inner membrane ABC transporter FepC_2_D_2_ [53]. Moreover, *E. coli* synthesizes another ABC transporter, responsible for the transport of iron citrate through the inner membrane [54]. Similarly to FbpABCD from *Nm*, the FecBCDE inner membrane transport system transports iron Fe^3+^ but not the iron citrate complex [54]. In *E. coli*, FecBCDE can be hypothesized to also transport iron pyrophosphate. In *E. coli*, expression of the *fec* operon containing the structural genes of this inner membrane transport system is repressed by iron-loaded Fur and induced in the presence of iron-loaded citrate [55]. In the absence of iron-loaded citrate, basal expression of the *fecABCDE* operon would not be sufficient to promote a speculated FecBCDE-dependent transport of iron pyrophosphate through the inner membrane. Within the cytoplasm, intracellular pyrophosphatase can degrade pyrophosphate and facilitate iron release. In addition, reduction of iron by a ferric reductase [56], [57] could provoke its release from pyrophosphate and methylene diphosphonic acid. Iron reduction by a reductase was reported to be responsible for iron release from siderophores like coprogen and ferrioxamine [58].

Our work opens up new insights into iron acquisition in *Nm.* Indeed, *Nm* seems to preferentially use iron among the transition metals. Several systems have been selected to allow highly efficient iron acquisition in the natural habitat of *Nm*. Pyrophosphate could permit iron acquisition from a wide range of iron sources like lactoferrin at sites such as the nasopharynx, the natural habitat of *Nm,* and might support meningococcal growth when in competition with other microbial species that produce siderophores, exhibiting lower affinity for iron than enterobactin. Thus, the presence of pyrophosphate enables Nm to obtain iron using a simple, highly competitive pathway.
